# Bacterial and fungal organisms in otitis externa patients without fungal infection risk factors in Erzurum, Turkey

**DOI:** 10.1016/S1808-8694(15)30524-3

**Published:** 2015-10-18

**Authors:** Murat Enoz, Irfan Sevinc, Jose Florencio Lapeña

**Affiliations:** 1 Dr. (Specialist); 2 VMD (Med. Microbiol., Department of Microbiology, Maresal Cakmak Military Hospital, Erzurum, Turkey); 3 MA, MD (Associate Professor, Department of Otorhinolaryngology, College of Medicine, Attending Otolaryngologist, Philippine General Hospital, University of the Philippines Manila) Department of ORL & Head and Neck Surgery, Maresal Cakmak Military Hospital, Erzurum, Turkey

**Keywords:** causative agents, polymicrobial, otitis externa, microbiology

## Abstract

**Aim:** To describe the bacterial and fungal organisms in otitis externa patients without other risk factors for fungal infections.

**Study design:**

Cross sectional cohort descriptive study.

**Materials and Methods:**

Ear swabs were obtained from 362 patients aged 1 to 55 years old with clinically diagnosed otitis externa in Erzurum, Turkey, between January 2006 and April 2007, and cultured for aerobic and anaerobic bacteria and fungi, using EMB, 5% sheep's blood, chocolate agar, anaerobic blood agar plate, thioglycollate broth and sabaroud agar using standard microbiological technique to diagnose isolates.

**Results:**

219 cultures were positive and a total of 267 isolates were obtained. Of the isolates, 68.16% (n: 182) were aerobic or facultative bacteria, 1.12 % (3) were anaerobic bacteria, 30.71 % (82) were fungi and 17.5 % (38) were polymicrobial infections.

**Conclusion:**

Fungal organisms especially Candida species may be isolated from ears of otitis externa patients without fungal infection risk factors such as ear self-cleaning, local antimicrobial, antifungal or corticosteroid drops or systemic antimicrobial or antifungal agents within the preceding week. Bacterial and fungal cultures may be recommended, and anti-fungal agents may be added, to treatment regimens in patients with otitis externa.

## INTRODUCTION

Otitis externa is a generic term for inflammation of the external auditory meatus (EAM) skin, which includes not only the visible ear but also the portion of the ear canal that leads up to the eardrum without extending to the middle ear. Usually infectious (bacterial or fungal) in etiology, the main symptoms include severe otalgia, purulent discharge and variable hypoacousia^1^. A recent increased incidence of fungal otitis may be due to overgrowth associated with the use of systemic broad-spectrum antibiotics^2^ and increased use of topical fluoroquinolone antibiotics^3^, ^4^, ^5^, ^6^.

Previous reports have questioned whether fungal organisms identified on culture represent colonization or infectious agents^3^, ^4^, ^5^, ^6^, ^7^. Among fungi, Aspergillus species are the predominant organisms implicated in the etiology of otomycosis^7^, ^8^.

Our study aimed to investigate the bacterial and fungal agents present in ear swabs of patients with otitis externa and an uneventful medical history.

## MATERIALS AND METHODS

With Institutional Review Board approval and individual informed consent, ear swabs were obtained from 362 patients diagnosed with otitis externa at the Maresal Cakmak Military Hospital, a secondary-care institution in Erzurum, Turkey, between January 2006 and April 2007. There were 124 males and 238 females with ages ranging from 1 to 55 years (average age: 26 years).

Inclusion criteria were a history of ear pain and / or itching and physical findings of erythema and swelling of the external auditory meatus (EAM) skin, and varied discharge (scanty white mucoid, grey, bluish-green and yellow discharges) or moist debris from the EAM lasting less than seven days. The diagnosis of otitis externa was made by a Resident Otolaryngologist (single observer). Excluded from the study were immunocompromised patients with Human Immunodeficiency Virus (HIV) infection, Diabetes Mellitus, eczema, ear self-cleaners and those treated with local antimicrobial, antifungal or corticosteroid drops or systemic antimicrobial or antifungal agents within the preceding week. The patients with diagnosed by resident otolaryngologist (single observer) as fungal infection of the ear canal with shows very characteristic findings such as black or white mass of the fungus (spores or hyphae of the fungus) also excluded from the study. Demographic data, history, symptoms and predisposing factors were recorded for each patient.

The cavum conchae and external meatus were cleaned with alcohol (70% isopropanol) before the application of swabs. Three specimens each of the affected external canal were obtained with three separate sterile cotton swabs with precautions taken to avoid contact with the cavum concha and external meatus. One swab was processed for aerobic bacteria in Stuart medium (Diomed) transport system, one swab was placed in an anaerobic thioglycolate medium, and one swab was placed in a sabaroud dextrose broth with antibiotics. Specimens were inoculated within 60 minutes into 5% sheeps blood, chocolate, and EMB agar plates for aerobic and facultative organisms and sabaroud dextrose agar with antibiotics (ASDA) for fungal organisms. The plates were incubated at 37°C aerobically (EMB) or under 5% CO_2_ (5% sheeps blood and chocolate) and examined at 24 and 48 hours. For anaerobes, the material was inoculated within 60 minutes into thioglycolate broth and plated onto an anaerobic blood agar plate containing kanamycin and vancomycin. These were incubated in GasPak jars (BBL) and examined at 48, 96 and 120 hours. Fungal cultures were evaluated for isolation of any fungal growth on ASDA after incubation at 25-26°C for 2 weeks. Standard microbiological technique was used in diagnosis of isolates.

For statistical analyses arithmetic mean and X2 tests were used and a values were chosen as p=0.01 and p=0.05.

## RESULTS

A total of 219 cultures were positive and a total of 267 isolates were obtained from 362 patient's ear swabs. Of the isolates 68.16% (182) were aerobic or facultative bacteria, 1.12 % (3) were anaerobic bacteria and 30.71 % (82) were fungi. The distribution of isolates in culture positive cases is summarized in Table I. The distribution of polimicrobial isolates in otitis externa is summarized in Table II.

**Table I:** Distribution of isolates in culture positive cases.

MICROORGANISM	No	%
BACTERIA		
Staphylococcus aureus	65	24,34
Streptococcus pyogenes	13	4,87
Streptococcus pneumoniae	16	5,99
Pseudomonas aeroginosa	32	11,99
Klebsiella pneumoniae	16	5,99
Klebsiella oxytoca	7	2,62
Klebsiella ozanae	4	1,50
Enterobacter aerogenes	11	4,12
Escherichia coli	6	2,25
Proteus mirabilis	6	2,25
Proteus vulgaris	2	0,75
Moraxella catarrhalis	4	1,50
TOTAL AEROBIC-FACULTATIF BACTERIA	182	68,16
Bacteroides fragilis	2	0,75
Peptostreptococcus sp.	1	0,37
TOTAL ANAEROBIC BACTERIA	3	1,12
TOTAL BACTERIA FUNGI	185	69,29
Candida sp.	33	12,36
Aspergillus flavus	13	4,87
Aspergillus fumigatus	4	1,50
Aspergillus niger	9	3,37
Mucor sp.	10	3,75
Penicillium sp.	12	4,49
TOTAL FUNGI	82	30,71
TOTAL ISOLATES	267	100

**Table II:** Distribution of isolates in polymicrobial otitis externa

MICROORGANISM	No	%
Staphylococcus aureus + Pseudomonas aeroginosa	9	4,11
Staphylococcus aureus + Streptococcus pyogenes	1	0,46
Staphylococcus aureus + Klebsiella pneumoniae	4	1,83
Staphylococcus aureus + Enterobacter aerogenes	4	1,83
Staphylococcus aureus + Candida sp.	11	5,02
Proteus mirabilis + Candida sp.	2	0,91
Staphylococcus aureus + Penicillium sp.	3	1,37
Staphylococcus aureus + Aspergillus flavus	4	1,83
TOTAL	38	17,35

The polymicrobial nature of otitis externa in 17.35 % (n: 38) of the cases was reflected in this study. Fungal agents were found in 9.13 % (n: 20) of the cases as polymicrobial and in 21.58 % (n: 62) of the cases as solely cultured agents.

## DISCUSSION

Our study aimed to investigate the possible bacterial and fungal agents in patients diagnosed with otitis externa. Significant differences in isolation frequency were found between Staphylococcus aureus, Pseudomonas aeroginosa, Candida sp., and other microorganisms (P<0.01). Although bacterial agents isolated were compatible with the literature, Candida species were the most common fungal agents. Staphylococcus aureus is cited as the most common causative infectious agent^9^ and fungi are occasional pathogens in otitis externa, mainly in chronic infections^10^. Aspergillus spp., the main pathogens of otomycosis, are responsible for 54-80% of cases^11^,^12^. In systemic or disseminated fungal infections, it has been observed that systemic or local immunosuppression, exogenous materials (tympanostomy tubes), and systemic or local antibiotic treatment can serve as the major predisposing factors for otomycosis^10^, ^11^, ^13^.

C. albicans remains the most common opportunistic fungal agent which produces a wide clinical spectrum in humans as well as animals^14^, ^15^. It is more commonly found in temperate zones while Aspergillus niger is more common in tropical countries^16^.

Jadhav et al.^16^, Erkan et al.^17^ and Jaiswal et al.^18^ reported incidences of Candida Albicans in otomycosis respectively as 2.38%, 1.62% and 1.72% in their studies. In our study, we found a 12.36% incidence of Candida species in patients who had no underlying disease. Candida species is one of the floras of the human skin^16^, ^17^, ^18^. Thus, positive culture of the Candida species does not always mean that Candida is the pathogen of the otitis externa therefore; our study cannot differentiate between fungal colonization and fungal infection in polymicrobial cases. However, in our study 21.58 % (n: 62) of the cases fungi were found as solely cultured agents. This finding may be explained that Candida species can be pathogen in with otitis externa patients who have not any fungal infection risk factors.

Jaiswal^18^ from India and Sheikh et al.^15^ from Iran reported C. albicans as a predominant etiological agent of otomycosis. The higher likelihood of finding Candida species can be correlated with physical findings commonly associated with fungal infection. Candida species are commonly associated with thick, white fluid. In contrast, black hyphae visible in Aspergillus infections provide obvious clues to fungal infection. In our study, the patients with these characteristic findings of fungal infection are excluded from the study. The appearance of these physical findings, in the setting of a draining ear that has not resolved with standard treatment would benefit from both ear cultures and treatment directed at fungal organisms^19^.

In a similar study, Pino Rivero et al.^20^ found that Pseudomonas, mainly P. Aeruginosa (46.83%) followed by Staphylococcus (18.98%) were the most frequent causative agents for otitis externa, but identified strains of associated fungi in almost one fourth of the cases.

Amigot et al.^21^ isolated Candida from 9.7%, Aspergillus from 6.5%, associations of Aspergillus and Candida from 2.2%, Pseudomonas aeruginosa from 18.6%, Proteus mirabilis from 10.9%, Staphylococcus aureus from 10.9% and three associations of Pseudomonas aeruginosa and Proteus mirabilis from 1.4% of patients with otitis externa.

Loh et al.^22^ reported that coagulase-negative Staphylococcus and Aspergillus niger were the most common bacteria and fungi cultured respectively in otitis externa patents. They emphasized that self-cleaning of the ears was the most common predisposing factor.

Zaror et al.^23^ investigated the clinical characteristics and the predisposing factors for 20 otomycosis cases during one year. They found that the most frequent species were Aspergillus niger (35%) and Candida albicans (20%), the genus Aspergillus represented 75% of the isolates. However, they emphasized that lack of cerumen (70%) chronic otitis (30%) previous antibiotic therapy and eczema (25%) were the most outstanding predisposing factors for otomycosis.

Our study excluded patients with fungal infection risk factors, yet found that candida species were the most common cultured fungal agents in acute otitis externa.

## CONCLUSION

Otorrhea due to fungal organisms especially Candida species must be kept in mind as a possible causative agent in otitis externa patients without risk factors for fungal infection. Bacterial and fungal cultures may be recommended, and the treatment of these patients may require addition of antifungal drugs.
